# Salt Solution Treatments Trigger Antioxidant Defense Response against Gray Mold Disease in Table Grapes

**DOI:** 10.3390/jof6030179

**Published:** 2020-09-18

**Authors:** Khamis Youssef, Sergio Ruffo Roberto, Angélica Nunes Tiepo, Leonel Vinicius Constantino, Juliano Tadeu Vilela de Resende, Kamal A.M. Abo-Elyousr

**Affiliations:** 1 Agricultural Research Center, Plant Pathology Research Institute, 9 Gamaa St., Giza 12619, Egypt; 2 Department of Agronomy, Agricultural Research Center, Londrina State University, Londrina 86057-970, Brazil; leonel@uel.br; 3 Department of Animal and Plant Biology, CCB, Londrina State University, Londrina 86057-970, Brazil; angelicantiepo@gmail.com; 4 Department of Arid Land Agriculture, Faculty of Meteorology, Environment and Arid Land Agriculture, King Abdulaziz University, Jeddah 80208, Saudi Arabia; kaaboelyousr@agr.au.edu.eg; 5 Department of Plant Pathology, Faculty of Agriculture, University of Assiut, Assiut 71526, Egypt

**Keywords:** salt, mechanism of action, antioxidant enzyme, *Botrytis cinerea*

## Abstract

To obtain a clear understanding of the mode of action of potassium bicarbonate (PB), sodium silicate (SSi) and calcium chelate (CCh) solutions (1%) in inducing resistance to gray mold disease in table grapes, enzymatic and nonenzymatic investigations were carried out. In particular, changes in the activity of the enzymes superoxide dismutase (SOD), ascorbate peroxidase (APX) and peroxidase (POD), total phenolic content and total flavonoid content were studied. As indirect action, PB, SSi and CCh reduced the incidence of gray mold by 43%, 50% and 41%, respectively. The highest activity of SOD was detected at 48 h in SSi-treated tissue, PB-treated tissue and CCh-treated tissue, and it was 1.7-, 1.4- and 1.2-fold higher, respectively, compared to the control. The APX activity was significantly higher in SSi-treated tissue than in the control at 24, 48 and 72 h and showed an increase in activity 2-fold for all times. Additionally, PB, SSi and CCh increased the activity of POD by 1.4-, 1.2- and 2.7-fold at 48 h posttreatment, respectively. The results showed that CCh was the most pronounced salt to increase both total phenol and flavonoid contents by 1.3 and 2.1, respectively. Additionally, the three tested salts induced an increase in total phenols and total flavonoids at 48 h posttreatment. The obtained result is one more movement towards an overall understanding of the mechanism by which salt solutions act as antimicrobial agents against gray mold of table grapes.

## 1. Introduction

Gray mold disease caused by *Botrytis cinerea* Pers. ex Fr., can infect numerous fresh fruit and vegetables globally, including table grapes, causing severe losses in production and the profitability ratio [1]. Fungicides are the main methods to manage this disease in grapes and other horticultural commodities. Nevertheless, different restrictions, including legislation, resistance, issues of human health and environment, have restricted their application [2,3]. Thus, secure and protective alternative methods to manage this disease and its cause need to be expanded. Salts such as bicarbonate, silicate and chelate are grouped under items generally recognized as safe (GRAS) compounds by the United States Food and Drug Administration and have been used as disinfectants for several fruit crops. The antimicrobial activity of such salt solutions has been extensively described in vitro and in vivo, and their efficacy was proven against several plant diseases of various fruit and vegetables [4,5,6,7,8,9].

Unfortunately, information about the mode of action of these salt solutions is poor and remains basically unknown. Understanding the mode of action of such promising salts may help to optimize their use against plant diseases, particularly those attacking fresh horticultural crops. In a previous study carried out by our research group, the ultrastructural changes in *B. cinerea* treated with potassium bicarbonate (PB), sodium silicate (SSi) and calcium chelate (CCh) were investigated. In particular, their influence on morphological alternation by scanning electron microscopy and transmission electron microscopy and on reactive species of oxygen, mitochondrial membrane potential and adenosine triphosphate content was analyzed [10].

Plants such as grapes are frequently challenged by a diverse array of pathogenic microorganisms, but they are able to defend themselves against almost all attacks, and their protective mechanisms involve inducible defense reactions. Additionally, treating plants with various substances can induce local or systemic resistance to disease attack [11]. Some postharvest applications could prompt some mechanisms that influence the metabolic action of the treated products, such as activating the antioxidant mechanism of fruit and vegetables, resulting in enhancing the antioxidant status of such crops [12].

It is well known that plants with high doses of antioxidants have superior resistance to oxidative damage [13]. Peroxidases (POD, EC 1.11.1.7) play the main role during the synthesis of lignin, which acts as a cell wall reinforcement, improving resistance against several pathogens and altering the antioxidant ability of fruits to control pathogen infection [14]. Plant resistance to different stresses is often expressed by an increase in protective enzymes such as peroxidases. Thus, their activity is overexpressed when plant tissues are subjected to stresses such as low temperature, pathogen infection, UV radiation, poisonous gases or heavy metals. Superoxide dismutase (SOD, EC 1.15.1.1) is a primary enzyme in the defensive system against oxidative stress and is elicited to reduce the effect of superoxide radicals. SOD is well known to catalyze the dismutation of superoxide (O_2_^−^) to hydrogen peroxide (H_2_O_2_) and O_2_ and thus provide defense against oxidative stress [15]. Additionally, ascorbate peroxidase (APX, EC 1.11.1.11) is considered to be the second most important hydrogen peroxide-scavenging enzyme and is crucial for the protection of cell constituents from damage by H_2_O_2_ and hydroxyl radicals (^•^OH) [16]. In addition, nonenzymatic antioxidants are the other side of the antioxidant mechanism, including phenolics, flavonoids and others. Phenolics and flavonoids protect diverse components of the cell from damage and play an important role in plant growth and development by altering cellular processes [17]. Antioxidant enzymes such as SOD, APX and POD are associated with increased resistance [18].

To our knowledge, no specific study has been performed to investigate the ability of PB, SSi and CCh to induce natural host resistance via enzymatic and nonenzymatic antioxidants against gray mold of table grapes. The aim of this study was to determine the changes in the activity of superoxide dismutase (SOD), peroxidase (POD) and ascorbate peroxidase (APX), total phenolic content and total flavonoid content in grape tissues treated with salt solutions.

## 2. Materials and Methods

### 2.1. Effect of Salts on Inoculated Berries (Indirect Antifungal Activity of Salts)

Bunches of table grape cv. Italia (*Vitis vinifera* L.) were harvested at commercial maturity and placed into secured container boxes from a commercial vineyard located in Paraná State, Brazil. Grape bunches were selected for uniformity of size and appearance and absence of any diseases or disorders. Bunches were surface decontaminated with a 2% commercial bleach (sodium hypochlorite) solution for approximately 2 min, washed with tap water and air-dried at room temperature. Individual berries were wounded once with a sterile nailhead along the equatorial axis. For each treatment, a 5 μl aliquot of potassium bicarbonate (PB, KHCO_3_ 99.7–100.5%, Synth, Brazil), sodium silicate (SSi, Na_2_O 18% + SiO_2_ 63%, Dinamica, SP, Brazil) and calcium chelate (CCh, C_10_H_12_N_2_O_8_CaNa_2_.2H_2_O 98%, Vetec Quimica Fina, RJ, Brazil) solution at 1% was applied to each wound (2 mm wide × 2 mm deep). After 24 h of incubation at 23± 1 °C, another wound was made approximately 5 mm apart from the previous wound. This wound was inoculated with 5μL of a 10^6^ conidia mL^−1^ *B. cinerea* (BC-UEL-1) suspension, prepared according to Youssef et al. [19]. The control consisted of berries treated in the first wound with sterile distilled water and then inoculated with the pathogen conidial suspension in the other wound. Treated berries were placed in plastic boxes (20 × 13 × 10 cm) and incubated at 23 ± 1 °C and high humidity (90–95%). Each treatment contained three replicates with 30 fruits per replicate. The entire experiment was repeated twice. The incidence of decay (% infected wounds) and disease severity (lesion diameter, mm) were recorded after 4 days of incubation.

### 2.2. Plant Material, Crude Extract and Experimental Design

Bunches of table grape cv. Italia (*Vitis vinifera* L.) were harvested, surface decontaminated and air-dried at room temperature as described above. Samples were immersed for five min as follows: (i) bunches treated with water (control); (ii) bunches treated with potassium bicarbonate (PB) solution at 1%; (iii) bunches treated with sodium silicate (SSi) solution at 1%; (iv) bunches treated with calcium chelate (CCh) solution at 1%. For each treatment, berries were randomized and arranged into 4 lots for tissue excision at different time intervals (0, 24, 48, 72 h). Each lot was made up of 4 replicates of 60 berries. At the end of the experiment, berries were arranged in plastic boxes, individually wrapped into a plastic bag and maintained at 90–95% relative humidity and 20 °C for 72 h. At the established time intervals, grape tissues were excised. Tissues were excised using sharp blades at different time intervals (0, 24, 48, 72 h). The excised tissues were rapidly frozen in liquid nitrogen, mixed and ground to a fine powder and directly stored at −80 °C until further analysis. Frozen tissues were freeze-dried using a lyophilizer (Liotop, L101 connected to dry scroll pump Agilent IDP7) for the lack of hydration procedure, reaching a final pressure of 17 µHg and condenser temperature of −52 °C.

### 2.3. Defensive Enzyme Assays

#### 2.3.1. Sample Preparation

To determine the activity of antioxidant enzymes, 200 mg lyophilized grape tissues were homogenized with 2 mL of phosphate buffer (100 mM, pH 7.5) containing 1 mM EDTA and 2% (*w*/*v*) polyvinylpolypyrrolidone. The mixture was centrifuged at 15,000× *g* for 15 min. The procedures of the enzymatic extract were carried out at 4 °C. The aliquots of this extract were kept at −80 °C until the enzymatic assays. Protein content was determined using the Coomassie Blue reagent at 595 nm [20]. All spectrophotometric analyses were performed using a GENESYS 10S UV-Vis spectrophotometer (Thermo Fisher Scientific Inc., Waltham, MA, USA).

#### 2.3.2. Superoxide Dismutase (SOD) Enzyme Assays

The methodology of Giannopolitis and Ries [21] with minor modifications according to Tiepo et al. [22] was followed to determine SOD activity. The reaction mixture was prepared with phosphate buffer (50 mM, pH 7.8), methionine (13 mM), EDTA (0.1 mM), nitro blue tetrazolium (NBT, 75 µM), and riboflavin (2 µM), and 40 μL of enzymatic extract was added to obtain 2 mL of final volume. The reaction was carried out under light from three fluorescent bulbs (25 W each) for ten minutes. An identical solution for each sample that was not illuminated served as a blank. Two milliliters of the reaction buffer without the enzyme extract was illuminated and served as a control. Each unit was marked as the quantity of enzyme required to result in a 50% inhibition of the rate of nitroblue tetrazolium photoreduction determined at 560 nm.

#### 2.3.3. Ascorbate Peroxidase (APX) Enzyme Assays

APX activity was determined following the methods of Nakano and Asada [23], with minor modifications according to Tiepo et al. [22]. A total volume of 3 mL as the reaction mixture contained phosphate buffer (50 mM, pH 7.0), EDTA (0.1 mM), ascorbate (0.5 mM), H_2_O_2_ (30 mM) and 30 µL of enzyme extract. The H_2_O_2_-dependent oxidation of ascorbate was pursued by a decrease in the absorbance at 290 nm in kinetics mode (ε = 2.8 mM^−1^ cm^−1^).

#### 2.3.4. Peroxidase (POD) Enzyme Assays

POD activity was determined according to the method of Peixoto et al. [24], with minor modifications. The reaction phosphate buffer (25 mM, pH 6.8) was prepared containing pyrogallol (20 mM) and H_2_O_2_ (20 mM). One hundred microliters of enzyme extract were added to 1.9 mL of the reaction buffer. To stop the reaction after one minute, 200 µL of sulfuric acid was added to the reaction. The enzyme activity was determined at 420 nm to calculate purpurogallin formation (ε = 2.47 mM^−1^ cm^−1^).

### 2.4. Non-Enzymatic Assays

#### 2.4.1. Sample Preparation

A suspension was prepared from 1.0 g of each sample in 10 mL of 70% ethanol (*v*/*v*) for extraction of phenolic compounds and flavonoids. Then, the suspension was shaken for 2 h at 120 rpm and 30 °C. The extract was centrifuged at 1013× *g* (Excelsa 2 Fanem model 205N) for 5 min, and the supernatant was reserved for analysis [25].

#### 2.4.2. Total Phenolic Content

The total phenolic compound content was quantified spectrophotometry using the Folin-Ciocateau reagent [26], and absorbance was measured at 765 nm (Micronal, AJX1600). Gallic acid was used as a standard ranging from 0 to 100 mg L^−1^ (r = 0.9985), and the results were expressed as mg gallic acid equivalents (GAE) per 100 g DW.

#### 2.4.3. Total Flavonoid Content

The quantification of total flavonoids was based on Gurnani et al. [27], where quercetin was used as a standard ranging from 50 to 500 mg L^−1^ (r = 0.9987). The reaction medium contained NaNO_2_ (5%, *w*/*v*), AlCl_3_ (10%, *w*/*v*), NaOH (4%, *w*/*v*) and 250 μL of the ethanolic extract. The absorbance was measured at 425 nm, and the results were expressed as mg quercetin equivalents (QE) per 100 g DW.

### 2.5. Statistical Analysis

Experiments were repeated twice, and data were subjected to one-way analysis of variance using Statistica 6.0 software (Tulsa, OK, USA). To normalize variance, percentage data were arcsine-square root transformed before ANOVA analysis. Mean values were compared using Fisher’s protected LSD test and judged at the *p* ≤ 0.05 level. In linear graphs, data ± standard error of the mean was mentioned.

## 3. Results

### 3.1. Effect of Salts on Inoculated Berries (Indirect Antifungal Activity of Salts)

**Figure 1:**
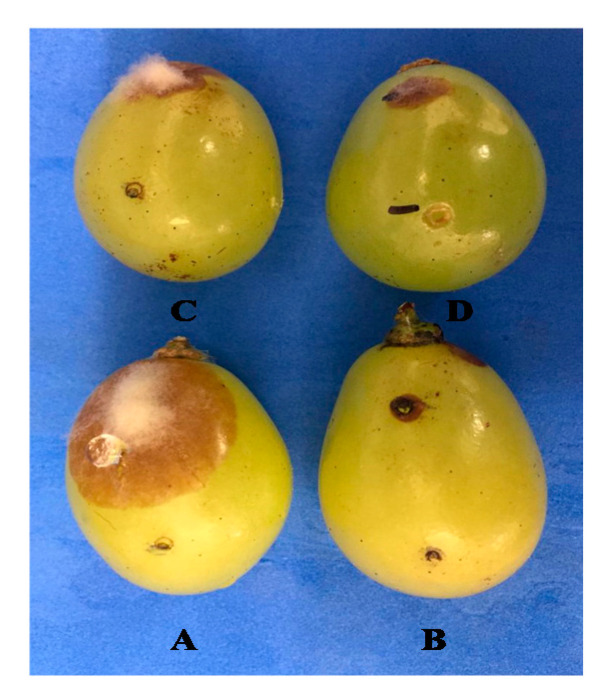
Indirect antifungal activity of PB, SSi and CCh at 1%. Salt solution and pathogen were added into separate wounds, (**A**): control; (**B**): PB; (**C**): SSi and (**D**): CCh.

**Figure 2:**
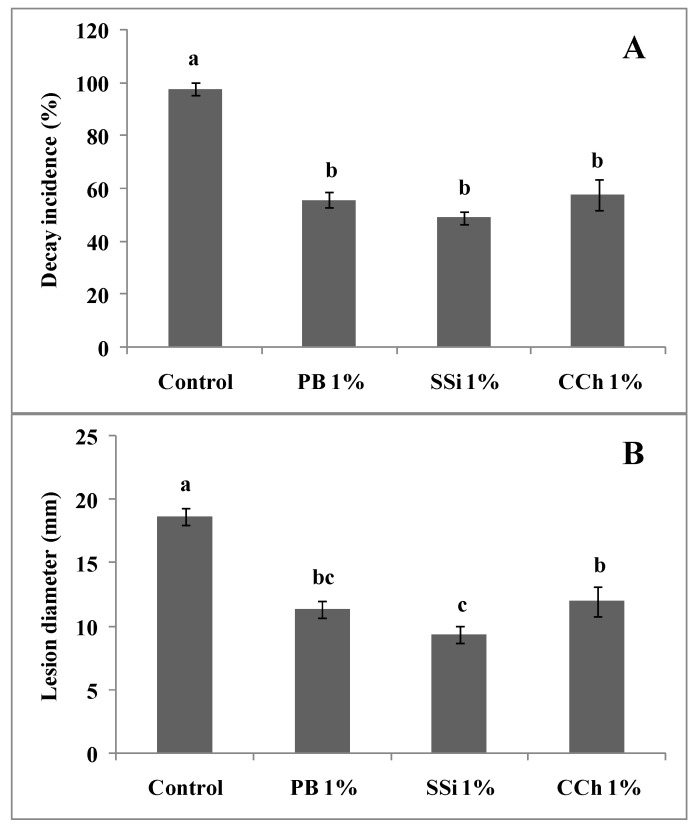
Decay incidence (**A**) and lesion diameter (**B**) of indirect antifungal activity of PB, SSi and CCh at 1%. Salt solution and pathogen were added to separate wounds. Columns marked with the same letters are not significantly different by Fisher’s protected LSD test at *p* ≤ 0.05.

In the case of salt and pathogens added to separate wounds, the reduction in gray mold incidence was 43%, 50% and 41% for PB, SSi and CCh, respectively. The lesion diameter of the disease in berries treated with PB, SSi and CCh was decreased by 39%, 50% and 36%, respectively, compared to berries not treated (Figure 1 and Figure 2).

### 3.2. Defensive Enzyme Assays

#### 3.2.1. SOD Enzyme Assays

**Figure 3:**
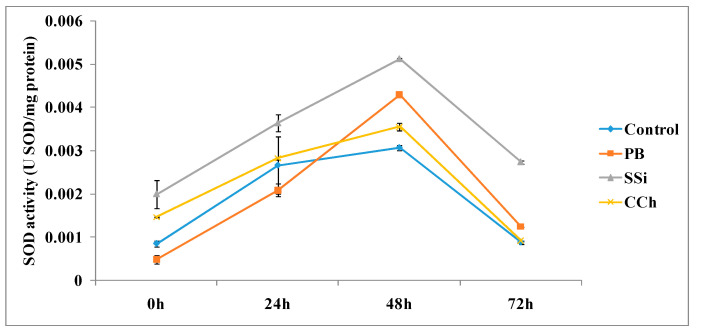
Time course of SOD activity (U SOD/mg protein) in extracts from table grape cv. Italia peel treated or not with PB, SSi and CCh. All the values are the means of three replicates ± SE.

The time course of SOD activities in table grapes cv. Italia treated with PB, SSi and CCh is shown in Figure 3. The SOD activity showed an increase with time in tissues treated with salts or water, reaching its maximum at 48 h in all treatments. The highest activity of SOD was detected at 48 h in SSi-treated tissue, PB-treated tissue and CCh-treated tissue, where it proved to be 1.7-, 1.4- and 1.2-fold higher, respectively, compared to the control. Additionally, SOD activity was significantly higher at 72 h in SSi-treated tissue and PB-treated tissue, resulting in 3- and 1.4-fold increases, respectively. However, no significant difference was observed for CCh-treated tissue compared to the control.

#### 3.2.2. APX Enzyme Assays

**Figure 4:**
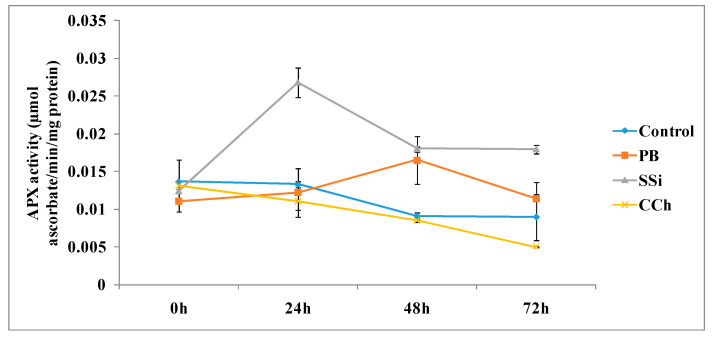
Time course of the APX activity (µmol ascorbate/min/mg protein) in extracts from table grape cv. Italia peel treated or not with PB, SSi and CCh. All the values are the means of three replicates ± SE.

The time course of APX activities in table grapes cv. Italia treated with PB, SSi and CCh is shown in Figure 4. The APX activity was significantly higher in SSi-treated tissue than in the control at 24, 48 and 72 h and showed an increase in activity 2-fold for all times. Additionally, APX activity proved to be higher at 48 h with an increase of 1.8-fold compared to the control treatment. However, CCh did not significantly change the activity of APX at all time course assessments.

#### 3.2.3. POD Enzyme Assays

**Figure 5:**
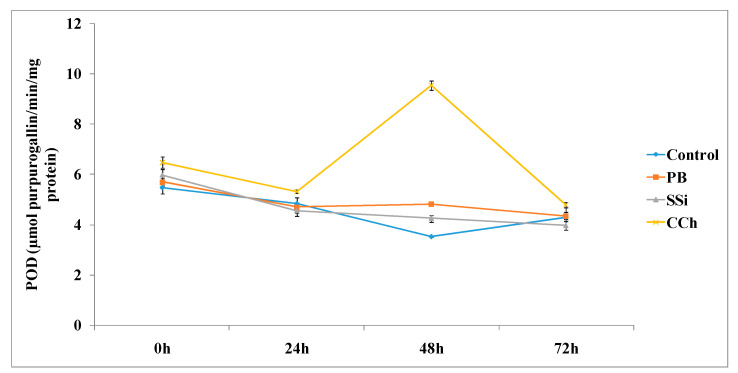
Time course of POD activity (µmol purpurogallin/min/mg protein) in extracts from table grape cv. Italia peel treated or not with PB, SSi and CCh. All the values are the means of three replicates ± SE.

The time course of POD activities in table grape cv. Italia treated with PB, SSi and CCh is shown in Figure 5. Overall, at 48 h, PB, SSi and CCh increased the activity of POD by 1.4-, 1.2- and 2.7-fold, respectively. In particular, the highest activity of POD was observed at 48 h in CCh-treated tissue, where it was 2.7-fold superior to the control. Additionally, no significant difference was noted at 24 and 48 h posttreatment.

### 3.3. Non-Enzymatic Assays

#### 3.3.1. Total Phenolic Content

**Figure 6:**
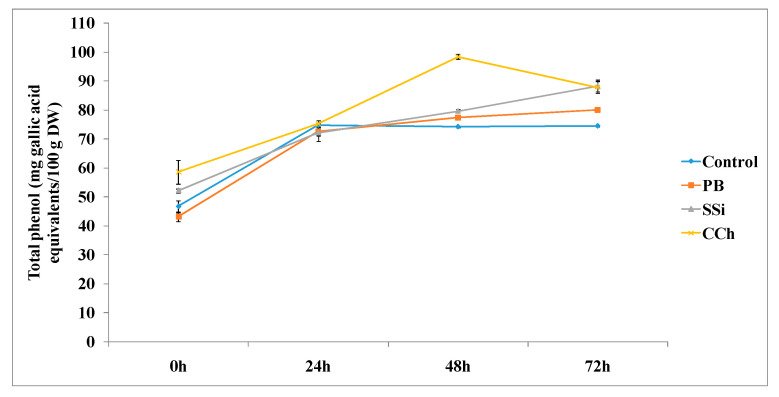
Time course of phenol content (mg gallic acid equivalents/100 g DW) in extracts from table grape cv. Italia peel treated or not with PB, SSi and CCh. All the values are the means of three replicates ± SE.

The total phenol content was determined at various time intervals, as shown in Figure 6. The three tested salts induced an increase in total phenol at 48 and 72 h posttreatment. In particular, the total phenol content was significantly higher in CCh-treated tissue, PB-treated tissue and SSi-treated tissue by 1.3-, 1.1- and 1.1-fold, respectively, at 48 h compared to the control. The same trend was also observed at 72 h posttreatment.

#### 3.3.2. Total Flavonoid Content

**Figure 7:**
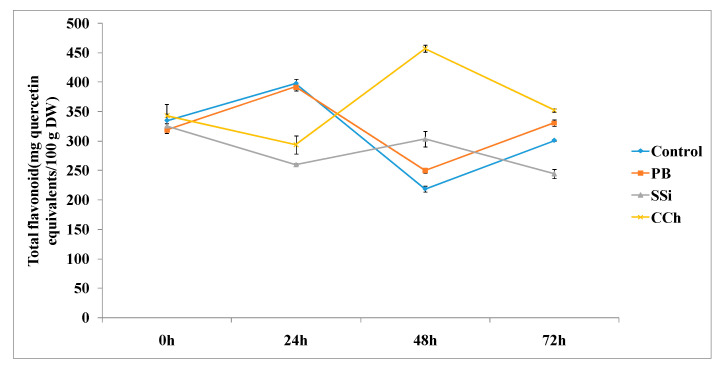
Time course of flavonoid content (mg quercetin equivalents/100 g DW) in extracts from table grape cv. Italia peel treated or not with PB, SSi and CCh. All the values are the means of three replicates ± SE.

The total flavonoid content was measured at different time intervals, as shown in Figure 7. PB, SSi and CCh induced a significant increase in total flavonoids by 1.2-, 1.4- and 2.1-fold, respectively, at 48 h posttreatment. At 72 h posttreatment, the total flavonoid content was higher in PB and CCh-treated tissues by 1.1- and 1.2-fold, respectively, whereas SSi showed a decrease in flavonoid content compared to the control.

## 4. Discussion

The main objective of this research was to verify the changes in the activity of enzymatic (SOD, POD, APX) and nonenzymatic antioxidants (total phenolic and flavonoid content) on table grape tissues treated with salt solutions (PB, SSi, CCh). The indirect antifungal activity (salt solution and pathogen suspension were placed into separated wounds) of PB, SSi and CCh significantly reduced the gray mold incidence of table grape cv. Italia by 43%, 50% and 41%, respectively, confirming the previous direct effect of those salts against *B. cinerea* (salt solution and pathogen suspension were placed together in the same wound) [10].

The results of this study revealed that the highest activity of SOD was detected at 48 h in SSi-, PB-and CCh-treated tissue at 48 h posttreatment, and this superior activity was maintained until 72 h in SSi-treated tissue and PB-treated tissue. It was mentioned previously that SOD catalyzes superoxide radicals to hydrogen peroxide and oxygen in a reaction that is spontaneous and very rapid, therefore protecting the cells from damage by superoxide radical reaction products. Our results are in agreement with those of Ogawa et al. [28], who observed a significant increase in SOD in leaves pretreated with sulfamethoxazole at 12 and 24 h [29]. SOD participated in the lignification process by activating the formation of hydrogen peroxide. The balance between POD and SOD activities in cells was vital to determine the steady-state level of oxygen and hydrogen peroxide [30]. Thus, treatments with PB, SSi and CCh activated protective enzymes, balanced the content of O_2_^−^ and H_2_O_2_ and induced resistance against gray mold of grapes.

The obtained results herein proved that SSi and PB induce an increment of APX activity by 2- and 1.8-fold when compared with untreated tissue (control) at 48 h. Above all, APX activity was significantly higher in SSi-treated tissue at 24, 48 and 72 h. It is well known that APX is a key antioxidant enzyme of scavenging systems. It catalyzes the conversion of hydrogen peroxide into water, employing ascorbate as an electron supporter. Enhanced peroxidase activity by more than 100% was observed in sodium-silicate-treated melons [31]. It is well known that POD plays a significant role in the defense systems of plants against fungal infections [32]. Our results indicate that CCh did not significantly change the activity of APX at all time course assessments. Previously, it was mentioned that the activity of APX rapidly loses stability and declines under conditions in which the ascorbate concentration is lower than 20 μM [33]. Additionally, to maintain an adequate cellular concentration of reactive oxygen species, APX isoenzymes play a protective role against ROS produced in excess under environmental stress [34]. In fact, a study carried out by our research group indicated that PB, SSi and CCh altered the morphology and function of mitochondria in *B. cinerea* and caused accumulation of ROS, reduced adenosine triphosphate content and decreased mitochondrial membrane potential, resulting in the loss of mitochondrial function [10]. APX plays an important role in the regulation of cellular ROS levels and is the central component of hydrogen peroxide-scavenging networks [35]. Similarly, in raspberry fruits, ethanol induced the accumulation of antioxidant compounds, inactivating ROS by antioxidative enzymes such as APX, SOD and CAT, and improving the antioxidant profile of crops [12]. Generally, alterations in the antioxidative defense system have been shown in various horticultural crops exposed to UV radiation, which increase the activities of antioxidant enzymes, including APX, in strawberry during storage [36].

The results obtained in this research indicated that PB, SSi and CCh increased the activity of POD by 1.4-, 1.2- and 2.7-fold, respectively, at 48 h posttreatment and that CCh was the most pronounced salt. Similarly, sodium bicarbonate and carbonate increased the activity of POD in citrus-treated tissue at 24 and 12 h posttreatment, respectively [37]. As a consequence, fruit might be less susceptible to fungal invasion because of cell wall reinforcement (lignin formation) and increases in tissue antioxidant ability [38]. The results obtained herein are in agreement with other findings, which demonstrate increased POD activity in citrus fruits elicited by UV irradiation [39] and in response to *Penicillium digitatum* infection [14]. The obtained results suggested that POD has a role in the positive effect of postharvest salt application. Hyperactivities of POD by chemical elicitors have been documented in a variety of plant species, including pear, tomato, turmeric and cucumber [38,40,41,42]. Increased POD activity may participate in suberization and lignification of the host cell wall to restrict disease development, as reported earlier in tomato plants. Thus, the increase in POD is one of the markers of induced resistance.

Similarly, POD activity reached the maximum level at 48 h and was markedly induced in grapes treated with ammonium molybdate and *Hanseniaspora uvarum* alone or in combination [43]. It is known that POD is involved in the lignification and wound healing processes, and these processes are connected with pathogen penetration prevention into fruit tissues [44]. Additionally, it has been reported that 1% chitosan treatment of grapes increased the activities of the antioxidant enzymes SOD and POD compared to the control [45].

Regarding total phenol and flavonoid content, the results obtained herein showed that CCh was the most pronounced salt to increase both total phenol and flavonoid contents by 1.3- and 2.1-fold, respectively. Additionally, the three tested salts induced an increase in total phenol and total flavonoid at 48 h posttreatment. The results obtained herein are in agreement with previous studies indicating that chitosan treatment showed higher POD activity in table grape cv. El-Bayadi after storage compared to the control, and this activity increased as the chitosan rate increased. Additionally, chitosan at 1% retained higher total phenolic and total flavonoid levels and increased the antioxidant enzyme POD compared with the control of table grape cv. El-Bayadi [46]. Furthermore, higher enzymatic activities can lead to higher phenolic concentrations and enhanced antioxidant enzyme activities [47]. It is well known that POD controls the availability of hydrogen peroxide in the cell wall, which is a requirement for the cross-linking of phenolic groups in response to various external stresses [48]. In addition, treatment with UV-A or UV-B increased the levels of antioxidants such as ascorbate, α-tocophol and polyphenol [49].

The increased activity of antioxidant enzymes obtained by this research is in agreement with Ghorbani et al. [50], who showed that nitric oxide reduced decay on grape cv. Rish Baba and increased POD, APX and SOD activity, and this effect was correlated with enhanced antioxidant enzyme activities. Furthermore, on wine grapes, postharvest treatments of ozone and chitosan improved the activity of antioxidant enzymes such as SOD and APX during the dehydration process, preventing polyphenol loss and evading membrane oxidation [51]. Finally, there is still a need to understand how different plant tissues and their metabolic pathways respond to different salt solutions. Studying transcriptome profiling may provide more details to enhance our knowledge about the mechanism of action of salt solutions against postharvest diseases.

## 5. Conclusions

The induction of the enhanced activities of SOD, POD, APX, phenolic and flavonoid contents may be directly correlated with the mechanism by which salt solutions such as PB, SSi and CCh induce resistance against gray mold in table grapes. The obtained result is one more movement towards an overall understanding of the mechanism by which salt solutions act as antimicrobial agents against the gray mold of table grapes. It is concluded that enhancing the natural host resistance was related to the enhancement of the antioxidant system.
